# Open-Angle Glaucoma Presenting as Delayed-Onset Interface Fluid Syndrome in a Patient With Minimal Risk Factors

**DOI:** 10.7759/cureus.103169

**Published:** 2026-02-07

**Authors:** Matthew McHarg, Matthew Denny, Sunita Radhakrishnan

**Affiliations:** 1 Department of Ophthalmology, California Pacific Medical Center, San Francisco, USA; 2 Department of Ophthalmology, Glaucoma Center of San Francisco, San Francisco, USA

**Keywords:** interface fluid syndrome (ifs), lasik, lasik complications, lasik interface, open-angle glaucoma

## Abstract

A 24-year-old male underwent uncomplicated myopic laser-assisted in situ keratomileusis (LASIK) three years prior to presentation to our clinic for left eye blurry vision. On initial examination, fluid was noted at the LASIK flap interface, with an elevated intraocular pressure (IOP) in the left eye. His pressure remained high despite treatment with multiple topical IOP-lowering agents, and visual field and retinal nerve fiber layer (RNFL) testing showed glaucomatous damage. He was subsequently referred to a glaucoma specialist and underwent surgical implantation of a glaucoma drainage device in the left eye, with postoperative stabilization of vision and IOP. This case is unique due to the patient's age, the long latency period between LASIK and symptom onset, and the absence of typical predisposing risk factors for glaucoma or interface fluid syndrome (IFS). This report highlights the importance of regular IOP monitoring in postoperative LASIK patients and urges providers to have a low threshold for completing glaucoma testing in atypical cases of IFS.

## Introduction

Interface fluid syndrome (IFS) is caused by the accumulation of fluid between a corneal flap and stromal bed due to elevated intraocular pressure (IOP) after corneal refractive surgery. It is a well-known complication of laser-assisted in situ keratomileusis (LASIK), typically presenting early in the postoperative period in patients with elevated IOP and often in the context of steroid response or pre-existing glaucoma [1-3]. It presents with vision loss or blurry vision due to central fluid within the cornea. IFS typically resolves with IOP control and treatment of any underlying conditions; however, it can lead to glaucomatous progression if not addressed in a timely manner [4,5]. Additionally, IFS itself can cause falsely low IOP readings, making it difficult to detect glaucomatous optic nerve damage. Ophthalmologists must remain vigilant in monitoring for IOP response and IFS in post-refractive LASIK patients [6,7].

## Case presentation

A 24-year-old male presented with painless blurred vision in the left eye. He underwent uncomplicated bilateral myopic LASIK in Egypt at age 22 and has been stable without the need for corrective lenses. The patient had a normal postoperative course after LASIK and had been tapered off steroid eye drops more than two years prior to this presentation. He had recently seen another ophthalmologist due to intermittent, transient episodes of altered vision in the left eye that had persisted for one month. He stated that during these episodes, he experienced constriction of his left visual field lasting for a few seconds with self-resolution. At this visit, he was noted to have an IOP of 33 in the left eye by Goldmann applanation tonometry; he was diagnosed with ocular hypertension and started treatment with brimonidine-timolol eye drops twice daily in the left eye. Over the next two weeks, he developed constant blurry vision that prompted him to seek a second opinion at our clinic.

**Figure 1:**
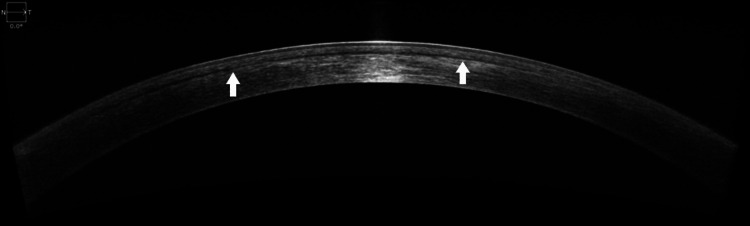
Anterior segment optical coherence tomography showing the presence of a thin hyporeflective band within the LASIK flap interface.

On our initial evaluation, best-corrected visual acuity (BCVA) was 20/20 in the right eye and 20/80 in the left eye. Applanation IOP was 16 in the right eye and 22 in the left eye, and he reported compliance with the topical therapy previously prescribed. Slit-lamp examination revealed a subtle fluid cleft in the LASIK flap interface of the left cornea with stromal haze. There was no flap displacement, epithelial ingrowth, or anterior chamber inflammation. Gonioscopy revealed open angles bilaterally, and there was no evidence of pigment dispersion or pseudoexfoliation. Anterior segment optical coherence tomography (AS-OCT) confirmed the presence of a hyporeflective band within the LASIK flap interface, consistent with IFS (Figure 1).

He had no identifiable risk factors for his ocular hypertension, including personal or family history of glaucoma, trauma, or recent steroid use. At this point, additional IOP-lowering therapies were added, including dorzolamide eye drops twice daily and latanoprost eye drops nightly in the left eye.

**Figure 2:**
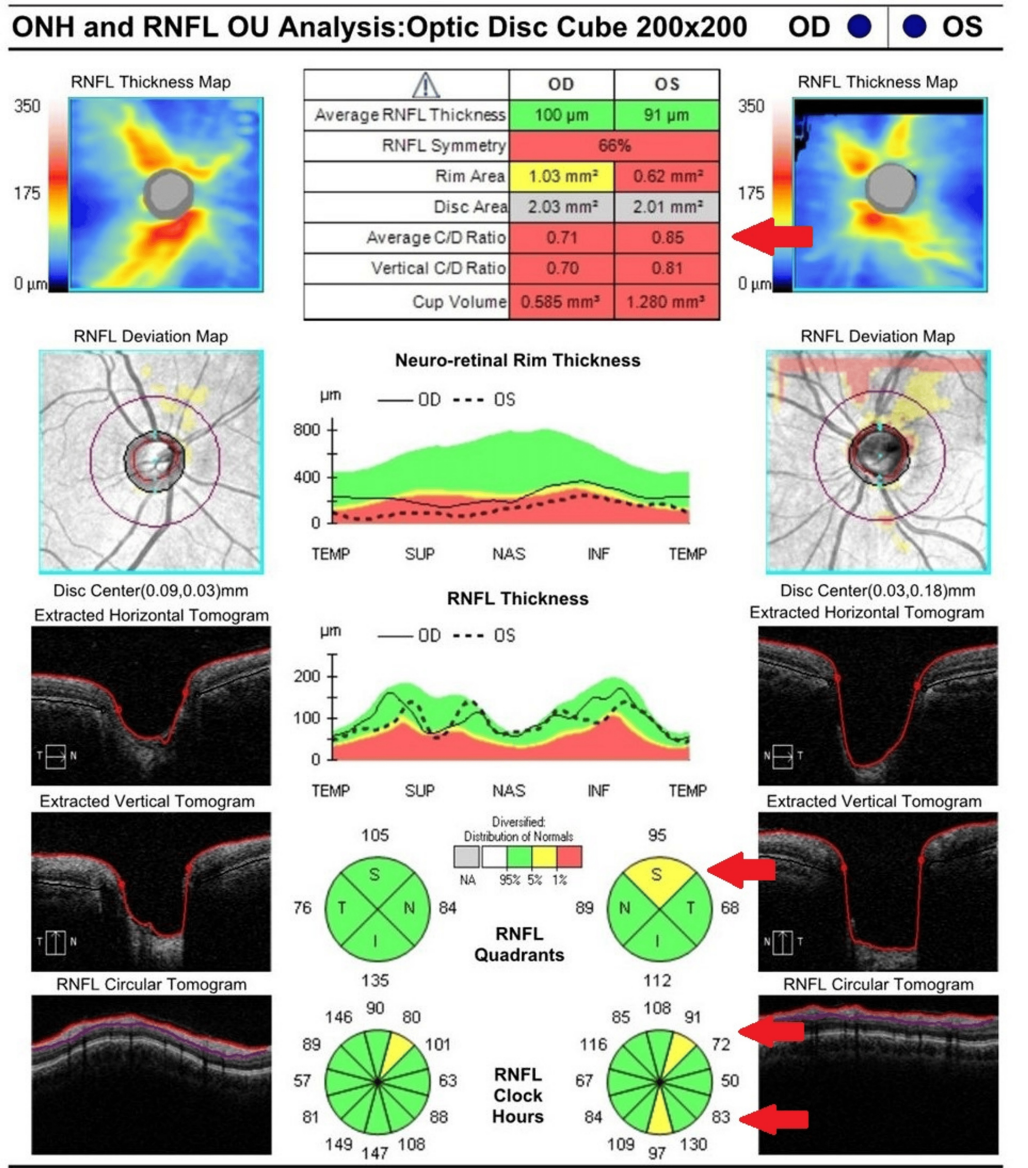
Initial retinal nerve fiber layer analysis showing superior and inferior thinning of the left eye (highlighted by red arrows). RNFL: retinal nerve fiber layer; ONH: optic nerve head

**Figure 3:**
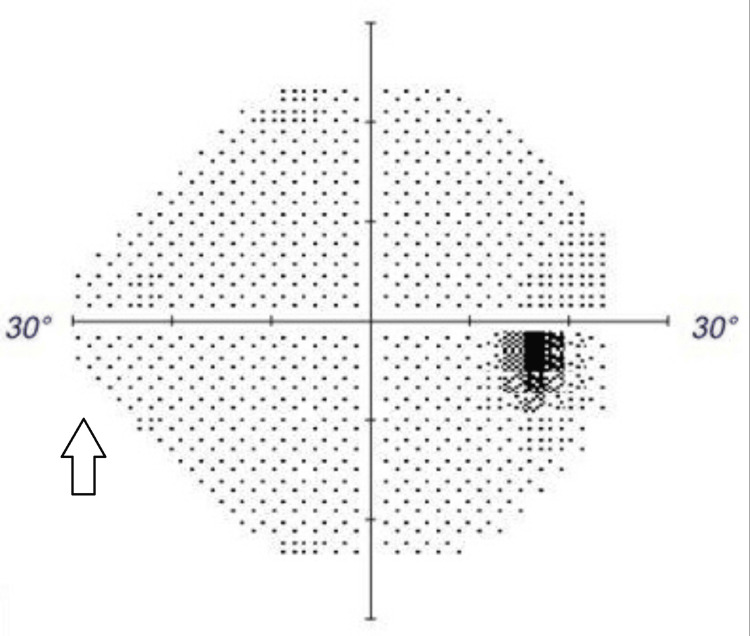
Humphrey Visual Field 24-2 of the right eye demonstrating a normal visual field.

**Figure 4:**
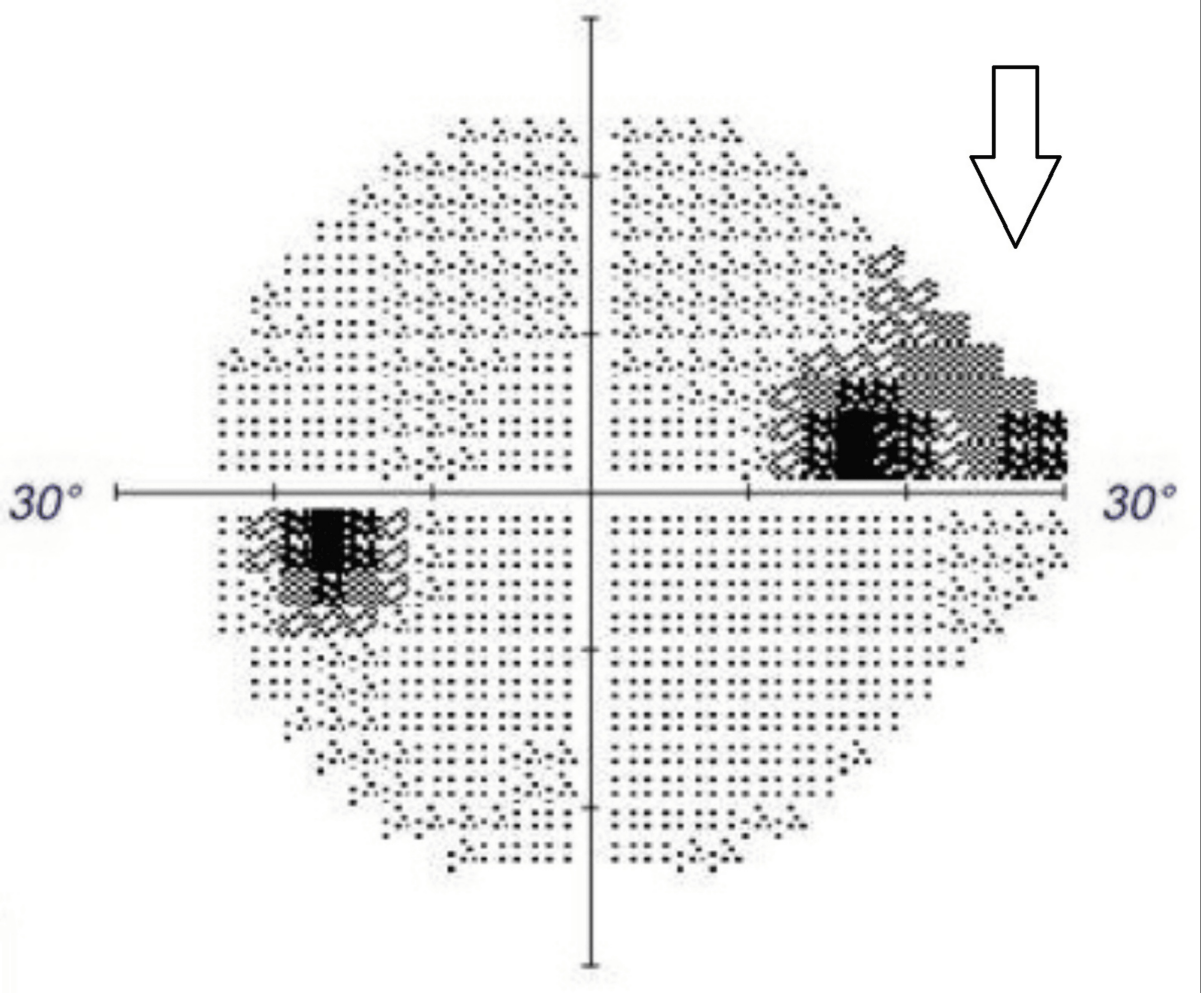
Humphrey Visual Field 24-2 of the left eye demonstrating a superonasal visual field deficit (highlighted by white arrow).

The patient returned to the clinic two weeks later and was found to have improved BCVA to 20/50. IOP remained stable at 23 in the left eye, and there appeared to be improvement in the interface fluid on exam and AS-OCT. However, after another two weeks, his BCVA had declined again to 20/150, and IOP measured 20 in the left eye while using three pressure-lowering eye drops. Further testing showed an enlarged cup-to-disc ratio and thin retinal nerve fiber layer (RNFL) in the superior and inferior quadrants and a superonasal visual field defect in the left eye (Figures 2-4).

The right eye vision and exam remained normal. At this time, the patient was started on netarsudil, oral acetazolamide, hypertonic saline, and oral valacyclovir. The valacyclovir was initially started for consideration of possible trabeculitis but was stopped three days later after being evaluated by a glaucoma specialist for further management.

**Figure 5:**
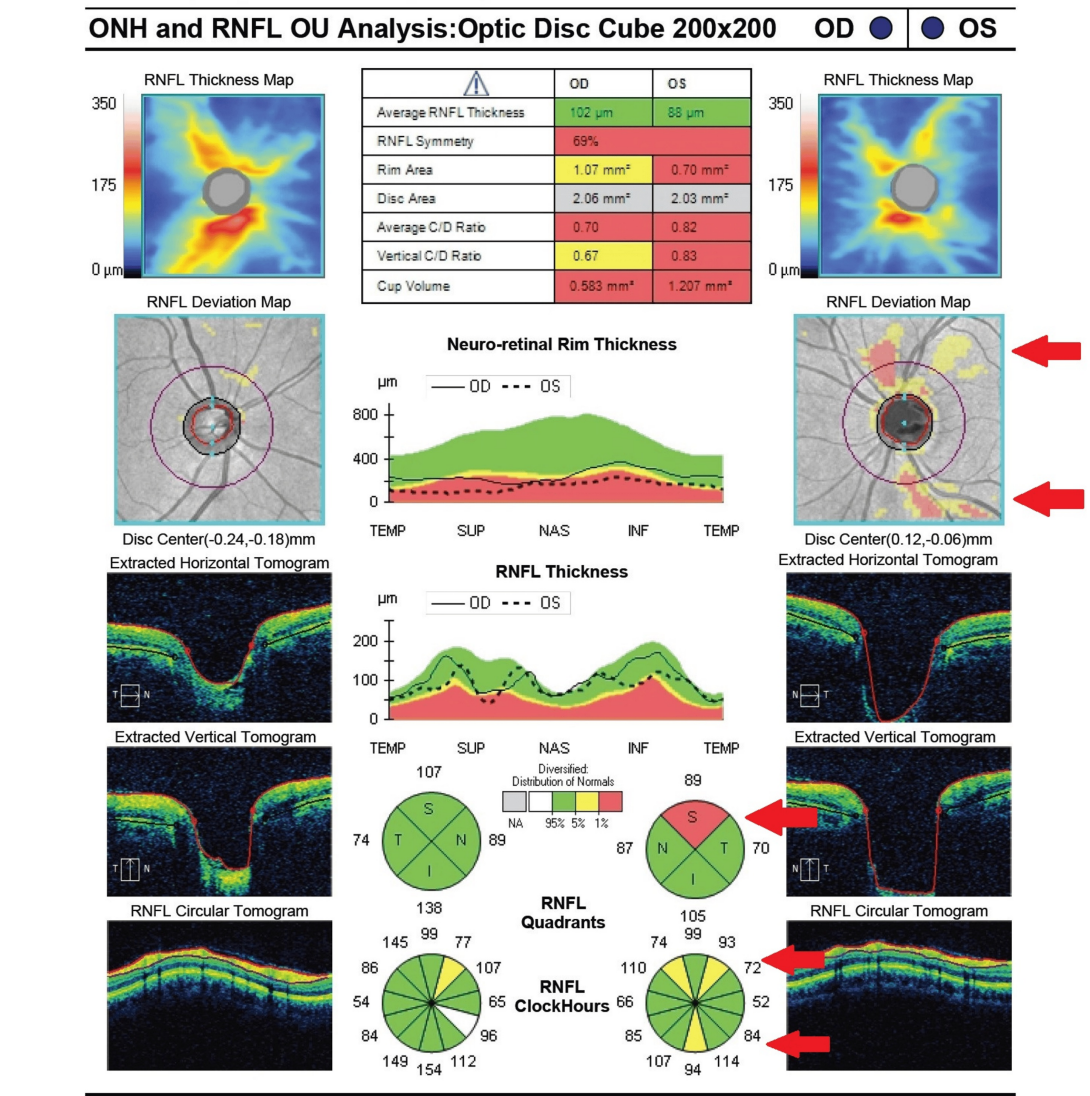
Repeat retinal nerve fiber layer analysis four weeks after initial presentation, showing worsening superior and inferior thinning of the left eye (highlighted by red arrows). RNFL: retinal nerve fiber layer; ONH: optic nerve head

On initial examination with the glaucoma specialist, BCVA was 20/300 in the left eye and improved to 20/40 with a pinhole. His applanation IOP was 24 at the central cornea and 30 at the peripheral cornea, with the patient looking up so the applanation tip was flush against the inferior cornea. There was corneal haze in the LASIK flap interface, and the anterior chamber was quiet. Gonioscopy was unchanged from the previous exam and showed normal, open angles in both eyes. Central corneal thickness was 470 in the right eye and 500 in the left eye, and on fundus examination, the cup-to-disc ratio was 0.7 in the right eye and 0.9 in the left eye. Repeat RNFL testing showed progression of superior and inferior thinning (Figure 5).

The patient had not started oral acetazolamide at this time, so he was advised to start taking this medication and continue all the prescribed topical IOP-lowering medications. He was also referred to a uveitis specialist, who performed an anterior chamber paracentesis and polymerase chain reaction (PCR) testing, which was negative for viral etiologies and *Toxoplasma*. With topical and oral glaucoma medications, his applanation IOP decreased to 17 in the left eye, measured at the central cornea, and 19 at the inferior peripheral cornea. His visual acuity improved only slightly, and he began to have intermittent episodes during which he described "completely clouded over" vision. He subsequently underwent Ahmed glaucoma valve implantation in the left eye, following which both IOP and visual acuity improved; the corneal edema and interface fluid resolved shortly after. At the last follow-up, seven weeks after surgery, BCVA was 20/80 in the left eye, with improvement to 20/20 on pinhole, and applanation IOP was 17 at the central cornea on brimonidine-timolol twice daily. Given the young age of onset of glaucoma and the absence of typical risk factors, the patient was also referred to a genetics clinic.

## Discussion

IFS is a well-known complication of LASIK surgery that has been reported in up to 2.9% of high-risk populations [2]. Risk factors include steroid-induced IOP increase, underlying glaucoma, and endothelial cell dysfunction. The typical timeline for IFS is in the early postoperative period - days to months after surgery [1,8]. Treatment aims to manage the IOP response and remove the interface fluid, usually with IOP-lowering agents and, if applicable, cessation of topical steroids [3,8].

This case is rare for several reasons, including the timing of disease presentation and the patient's young age without apparent risk factors for either glaucoma or IFS. There are previous case reports of delayed-onset IFS, but they are often in the context of an inciting event or with the presence of risk factors, including prolonged steroid use, underlying glaucoma, endothelial cell dysfunction, uveitis, additional ocular surgeries, trauma, or other infectious/inflammatory conditions [3-5,9-12]. These risk factors can manifest as IFS in post-LASIK patients when elevated IOP overwhelms the endothelial ability to regulate hydration, forcing fluid across the corneal stroma, which eventually leaks into the potential space created by LASIK flaps [7,8]. Our review of the literature found only two similar cases of late-onset IFS in young post-LASIK patients without significant risk factors; Mansoori described a young, otherwise healthy patient who experienced glaucomatous damage in both eyes in the setting of IFS, was lost to follow-up for a period of three years, and returned with worsening visual field loss requiring trabeculectomies in both eyes [13]. The second case reported by Koronis et al. was also similar to ours, except that their patient was started on steroid eye drops four days prior to being diagnosed with IFS [14].

Additionally, our patient had no obvious risk factors for developing ocular hypertension and glaucoma at such a young age. He did not have any features suggestive of secondary causes of elevated IOP, and there was no family history of glaucoma. Given the young age of onset, the presence of myopia, and the enlarged cup-to-disc ratio in the unaffected right eye, he may have juvenile open-angle glaucoma, which can present unilaterally in up to 25% of patients and is not always inherited in an autosomal dominant pattern [15]. He was referred for genetic testing, which is still pending. Research has shown that juvenile glaucoma patients typically have higher IOPs and a greater lifetime risk of perimetric blindness than other forms of open-angle glaucoma [16], so a positive genetic test would guide our patient's follow-up frequency and treatment plan. His genetic test results are also important in determining whether first-degree relatives or prenatal testing will be required in the future [17].

Lastly, the fluid in IFS alters the normal rigidity of the anterior cornea, resulting in falsely low applanation readings that can delay the identification and treatment of ocular hypertension and glaucoma [7,18]. Cabral-Macias et al. even went so far as to recommend peripheral IOP measurements for more accurate assessment in IFS patients, as peripheral IOP can be markedly different from central IOP due to the accumulation of interface fluid [7]. There is some variability in measuring peripheral IOP based on biomechanics such as corneal thickness, exact location of applanation, and curvature of the eye [19]; however, if completed by the same physician in a consistent manner, central and peripheral IOP measurements can be compared over time to determine a relative response to treatment. Our patient demonstrated higher IOP readings when measured peripherally, suggesting that his IOP may have been underestimated for the few weeks that he had IFS. However, the IOP had likely been elevated prior to this point, given his reported history of transient episodes of reduced vision prior to presentation and the extent of glaucomatous damage seen on RNFL and visual field testing completed just six weeks after flap interface fluid was first noted. This highlights the unpredictable course of IFS in glaucoma patients and the need to start treatment promptly.

## Conclusions

Providers should have a low threshold for offering glaucoma testing to patients with IFS. They should also be swift to escalate IOP-lowering treatment for patients with IFS, since it can mask ocular hypertension and/or underlying glaucoma for an extended period. This is a rare case of undetected open-angle glaucoma presenting as delayed-onset IFS in a patient without typical risk factors associated with IFS. Our patient highlights the potential difficulty in treating glaucoma patients who undergo LASIK and the increased likelihood of progression when these patients develop IFS. Post-LASIK protocols should include long-term follow-up with appropriate monitoring, including peripheral IOP measurements and OCT RNFL testing if IFS is suspected. These patients require prompt identification and treatment of post-surgical complications, including IFS.
